# The etiology and evolution of magnetic resonance imaging-visible perivascular spaces: Systematic review and meta-analysis

**DOI:** 10.3389/fnins.2023.1038011

**Published:** 2023-03-30

**Authors:** Serhat V. Okar, Fengling Hu, Russell T. Shinohara, Erin S. Beck, Daniel S. Reich, Benjamin V. Ineichen

**Affiliations:** ^1^Translational Neuroradiology Section, National Institute of Neurological Disorders and Stroke, National Institutes of Health, Bethesda, MD, United States; ^2^Department of Biostatistics, Epidemiology, and Informatics, Penn Statistics in Imaging and Visualization Center, University of Pennsylvania, Philadelphia, PA, United States; ^3^Department of Neurology, Icahn School of Medicine at Mount Sinai, New York, NY, United States; ^4^Department of Neuroradiology, Clinical Neuroscience Center, University Hospital Zurich, University of Zurich, Zurich, Switzerland; ^5^Center for Reproducible Science, University of Zurich, Zurich, Switzerland

**Keywords:** enlarged perivascular spaces, Virchow-Robin spaces, magnetic resonance imaging, etiology, etiopathogenesis, biomarker, systematic review, meta-analysis

## Abstract

**Objectives:**

Perivascular spaces have been involved in neuroinflammatory and neurodegenerative diseases. Upon a certain size, these spaces can become visible on magnetic resonance imaging (MRI), referred to as enlarged perivascular spaces (EPVS) or MRI-visible perivascular spaces (MVPVS). However, the lack of systematic evidence on etiology and temporal dynamics of MVPVS hampers their diagnostic utility as MRI biomarker. Thus, the goal of this systematic review was to summarize potential etiologies and evolution of MVPVS.

**Methods:**

In a comprehensive literature search, out of 1,488 unique publications, 140 records assessing etiopathogenesis and dynamics of MVPVS were eligible for a qualitative summary. 6 records were included in a meta-analysis to assess the association between MVPVS and brain atrophy.

**Results:**

Four overarching and partly overlapping etiologies of MVPVS have been proposed: (1) Impairment of interstitial fluid circulation, (2) Spiral elongation of arteries, (3) Brain atrophy and/or perivascular myelin loss, and (4) Immune cell accumulation in the perivascular space. The meta-analysis in patients with neuroinflammatory diseases did not support an association between MVPVS and brain volume measures [R: −0.15 (95%-CI −0.40–0.11)]. Based on few and mostly small studies in tumefactive MVPVS and in vascular and neuroinflammatory diseases, temporal evolution of MVPVS is slow.

**Conclusion:**

Collectively, this study provides high-grade evidence for MVPVS etiopathogenesis and temporal dynamics. Although several potential etiologies for MVPVS emergence have been proposed, they are only partially supported by data. Advanced MRI methods should be employed to further dissect etiopathogenesis and evolution of MVPVS. This can benefit their implementation as an imaging biomarker.

**Systematic review registration:**

https://www.crd.york.ac.uk/prospero/display_record.php?RecordID=346564, identifier CRD42022346564.

## 1. Introduction

**FIGURE 1:**
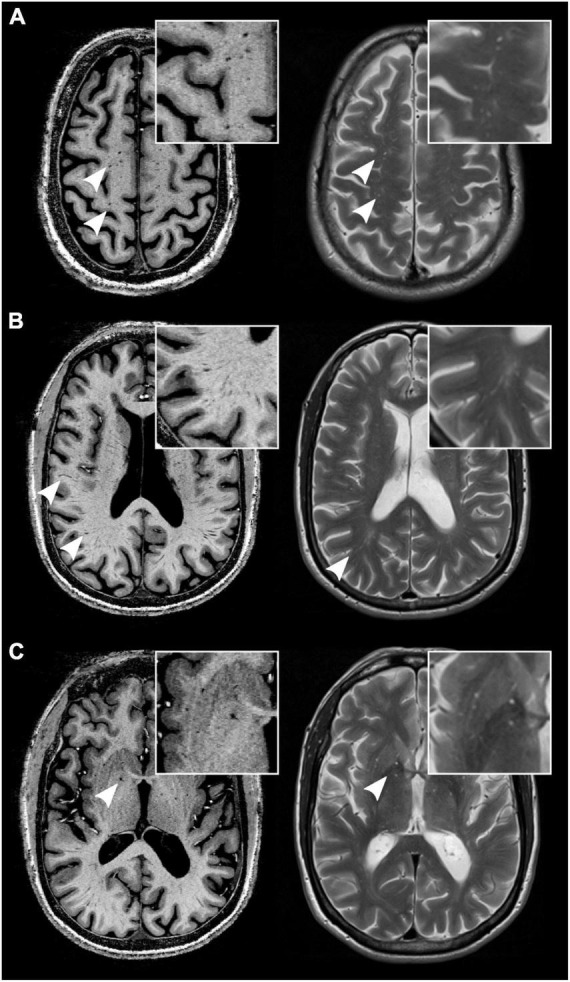
MRI-visible perivascular spaces (MVPVS) on magnetic resonance imaging (MRI). MVPVS are isointense to cerebrospinal fluid (CSF) on MRI on both T1 w (left column, acquired at 7-tesla static magnetic field strength) and T2 w/PD sequences (right column, acquired at 3-tesla static magnetic field strength) (white arrowheads). They commonly occur in the centrum semiovale **(A)**, deep white matter **(B)**, and basal ganglia **(C)**. Insets show higher magnifications. All images have an isotropic voxel size of 0.5 mm.

First described in detail by Virchow (1851) and confirmed as a feature of normal brain histology by Robin (1859), the perivascular space (PVS) is an anatomical compartment that follows the pial trajectories of brain vasculature, surrounding the arteries, veins, penetrating arterioles and venules (Troili et al., 2020; Wardlaw et al., 2020; Ineichen et al., 2022). Although PVS are a normal anatomic feature of brain vasculature that can be visualized using histology, they can also become visible on magnetic resonance imaging (MRI) if they exceed a certain diameter (depending on the resolution of the MRI). These macroscopically visible PVS have been referred to as enlarged PVS (EPVS), dilated PVS, or Virchow-Robin spaces (Ineichen et al., 2022) (Figure 1). In this review, we will use the term MRI-visible PVS (MVPVS) to acknowledge that temporal dynamics of PVS are insufficiently understood and thus to retain a more descriptive terminology.

On MRI, MVPVS show cerebrospinal fluid (CSF) signal characteristics, appearing as linear-shaped signal changes with a parenchymal vessel distribution (Wardlaw et al., 2013a). Typical locations of MVPVS are in the basal ganglia along lenticulostriate vessels, centrum semiovale, and midbrain (ponto-mesencephalic junction) (Kwee and Kwee, 2007). Less frequent MVPVS locations are: insula (Yamaguchi et al., 2021), hippocampus (Zhu et al., 2011), anterior temporal lobe (McArdle et al., 2020), corpus callosum (Manara et al., 2010, 2011), mesencephalon-thalamic junction (Salzman et al., 2005), and cerebellum (Alqahtani et al., 2014). Although many agree that MVPVS should be an imaging component of cerebral vessel disease (Francis et al., 2019), emerging data suggest that it can also be a feature of metabolic (Manara et al., 2011), neurodegenerative (Charidimou et al., 2017), and neuroinflammatory diseases (Granberg et al., 2020).

Since MVPVS can occur in a large spectrum of neurological and systemic diseases affecting the central nervous system (CNS), it is important to better understand MVPVS etiopathogenesis and their longitudinal evolution. Although some pathomechanisms, such as impaired interstitial fluid (ISF) drainage, have been proposed as MVPVS etiology (Wardlaw et al., 2013a,^2020^; Troili et al., 2020), the biological basis of MVPVS and longitudinal evolution of MVPVS in different diseases remain uncertain.

This systematic review and meta-analysis aims to answer the following two questions: (1) Which etiopathogeneses have been shown and/or proposed for MVPVS? (2) How do MVPVS evolve over time in healthy individuals or in individuals with CNS diseases?

## 2. Materials and methods

We registered the study protocol in the International prospective register of systematic reviews (PROSPERO, CRD42022346564)^1^ and used the Preferred Reporting Items for Systematic Reviews and Meta-Analysis (PRISMA) Guidelines for reporting (Moher et al., 2015).

### 2.1. Search strategy

We searched for original studies published in full up to December 12, 2022, in PubMed, Scopus, and Ovid EMBASE. The search string was created in PubMed and translated to the other databases. It contained two blocks: one with terms for enlarged perivascular spaces and one with terms for etiology or evolution of MVPVS, combined by the Boolean operator “AND”. We searched reference lists of included articles for additional eligible articles.

### 2.2. Inclusion and exclusion criteria

We included publications on human or animal data that reported on any outcome related to etiology and/or temporal dynamics of MVPVS. Reviews were included if they discussed MVPVS etiologies. We excluded conference abstracts, non-English articles, and publications that reiterated previously reported quantitative data.

### 2.3. Study selection and data extraction

Titles and abstracts of studies were screened for their relevance in the web-based application Rayyan by two reviewers (SO and BI) (Ouzzani et al., 2016), followed by full-text screening. Subsequently, the following data were extracted: title, authors, publication year, study design, disease, and number of included subjects as well as data on MVPVS definition, etiology, and temporal dynamics.

### 2.4. Quality assessment

The quality of each study with ≥10 included subjects was assessed against predefined criteria by two reviewers (SO and BI) using an adjusted version of the Joanna Briggs Institute Critical Appraisal Tools. Discrepancies were resolved by discussion.

### 2.5. Data synthesis and analysis

Only publications reporting correlation coefficients were included in the meta-analysis, and only summary-level data were used. We defined *a priori* that only diseases/conditions with more than three publications would be considered for a meta-analysis. Since MS and NMOSD are both neuroinflammatory entities, we decided post-hoc to pool MS and NMOSD studies for the meta-analysis to increase its statistical power. A random-effects model was fitted to the data. The amount of heterogeneity (i.e., τ^2^), was estimated using the DerSimonian-Laird estimator (DerSimonian and Laird, 1986). In addition, the *Q*-test for heterogeneity (Cochran, 1954) and the I^2^ statistic (Higgins and Thompson, 2002) are reported. A two-tailed *p*-value < 0.05 was considered statistically significant.

### 2.6. Publication bias

We defined *a priori* that we would assess publication bias if more than 10 studies were eligible for the meta-analysis. Thus, due to the limited number of studies eligible, we did not assess publication bias.

## 3. Results

### 3.1. Eligible publications and general study characteristics

#### 3.1.1. Eligible studies

**FIGURE 2:**
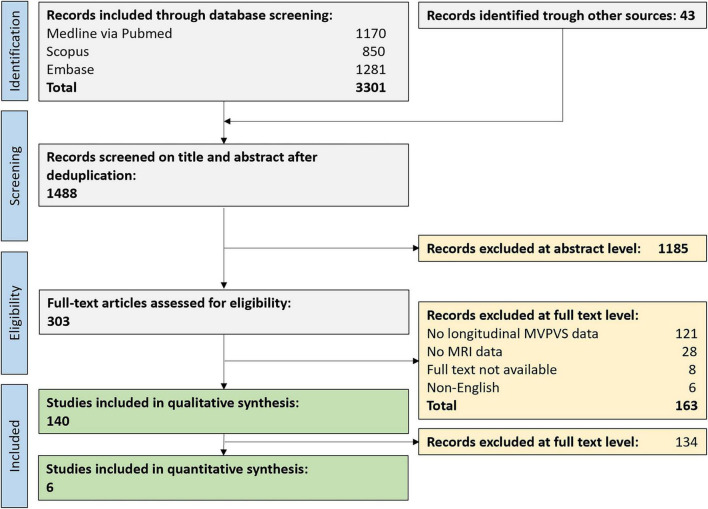
Flow chart of study inclusion. MVPVS, MRI-visible perivascular spaces.

In total, 3,301 original publications were retrieved from our comprehensive database search, and an additional 43 publications from reference lists of reviews on related topics. After abstract and title screening, 303 publications were eligible for full-text search. After screening the full text of these studies, 140 articles (9% of deduplicated references) were included for qualitative synthesis and 6 for quantitative synthesis (Figure 2). 109 publications assessed or discussed potential etiologies of MVPVS (including 19 reviews). 31 publications assessed longitudinal evolution of MVPVS. The median follow-up time of these studies was 36 months (range 1–204 months).

#### 3.1.2. Risk of bias assessment

Most of the publications reported the definition of MVPVS, mostly as being CSF-isointense longitudinal structures (91% of publications), and reported an MRI protocol (92%). Most publications also adjusted their analyses for age and sex (67%). Thus, the overall evidence was at low risk of bias for these domains.

### 3.2. MVPVS imaging and assessment methods

#### 3.2.1. Magnetic resonance imaging parameters

Eligible publications used a variety of different MRI parameters to visualize MVPVS. Applied static magnetic field strengths were between 1.5 and 7 tesla. Most commonly acquired images were T1-weighted (T1 w) and/or T2-weighted (T2 w), with a concomitant T2 w-FLAIR to distinguish MVPVS from other imaging features such as lacunes of presumed vascular origin (Wardlaw et al., 2013a).

#### 3.2.2. Assessment methods for enlarged perivascular spaces

There was a consensus to define MVPVS as linear to ovoid imaging features with an MRI signal intensity similar to that of CSF. Only one publication (Jiménez-Balado et al., 2020) particularly mentioned that MVPVS were assessed in accordance with the *Standards for Reporting Vascular Changes on Neuroimaging* (STRIVE) criteria (Wardlaw et al., 2013a). Eight publications did not report how MVPVS were defined. MVPVS were most commonly assessed in the centrum semiovale, the basal ganglia, and the brain stem.

Most studies assessed MVPVS using a manual scoring and/or segmentation. However, a couple of studies used automated segmentation methods using intensity-based thresholding approaches (Ramirez et al., 2015; Wang et al., 2016; Boespflug et al., 2018), vesselness filter approaches (Ballerini et al., 2018; Sepehrband et al., 2019), combination of these two methods (Spijkerman et al., 2022), or approaches based on machine-learning (Park et al., 2016; Hou et al., 2017; Boutinaud et al., 2021; Williamson et al., 2022) [reviewed in Moses et al. (2022)].

### 3.3. Etiology of MVPVS

We identified four partly overlapping hypothesized MVPVS etiopathogeneses:

#### 3.3.1. Hypothesis of interstitial fluid circulation impairment

**FIGURE 3:**
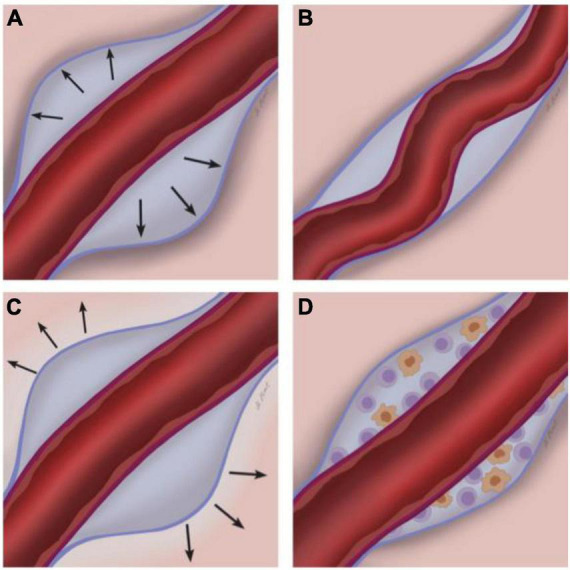
Potential etiopathogenesis of MRI-visible perivascular spaces (MVPVS). Four overarching and partly overlapping etiologies have been suggested for MVPVS: **(A)** Impairment of ISF circulation, potentially with abnormal blood-brain-barrier leakage (Fisher, 1979; Wardlaw et al., 2013b); **(B)** spiral elongation of vessels/tortuous vessels; **(C)** brain atrophy and/or perivascular myelin loss; and **(D)** immune cell accumulation in the perivascular space. The perivascular spaces are the compartments between the parenchymal basement membrane of the glia limitans (formed by compacted astrocyte foot processes and an overlying parenchymal basement membrane, blue) and the endothelial basement membrane of the blood vessel (purple) [reviewed in Ineichen et al. (2022)].

According to this proposed etiology, MVPVS emerge due to locally impaired ISF dynamics. Insufficient clearance of ISF via perivascular spaces could lead to fluid retention and thus enlargement of perivascular spaces (Figure 3A). Four partly overlapping mechanisms could lead to impaired ISF drainage via perivascular spaces.

##### 3.3.1.1. Vascular amyloid deposition hypothesis

Patients with cerebral amyloid angiopathy (CAA) are reported to harbor higher MVPVS burden (i.e., the overall extent of MVPVS) in the centrum semiovale compared to controls (Charidimou et al., 2013; Shams et al., 2016). This observation has been corroborated by data showing a spatial association between EPVS and CAA severity in the overlying cortex (Van Veluw et al., 2016; Perosa et al., 2022) which has led to the hypothesis that MVPVS could be a marker of impaired ISF drainage due to upstream amyloid deposits in the vessel wall. In fact, it has been suggested by DTI-ALPS (diffusion tensor image analysis along the perivascular space), an MRI-based approach (Taoka et al., 2017), that CAA is indeed associated with lower MVPVS diffusivity (Xu et al., 2022). However, by employing 11C-Pittsburgh B compound (PIB) PET (Banerjee et al., 2017) or florbetaben/florbetapir PET (Sepehrband et al., 2021; Wang et al., 2021; Jeong et al., 2022), the association between MVPVS and CAA has not been confirmed in the Alzheimer’s disease continuum, which commonly co-occurs with cerebral amyloid angiopathy. In addition, two studies found an association of MVPVS with tau by employing flortaucipir PET (Sepehrband et al., 2021; Wang et al., 2021) or CSF analysis (Wang et al., 2022). It is also noteworthy that it is still under debate whether MVPVS formation is the consequence or driver of vascular protein deposition (Lynch et al., 2022).

##### 3.3.1.2. Blood-brain-barrier leakage hypothesis

Small vessel disease could be a driver for the enlargement of perivascular spaces. This has been referred to as the blood-brain-barrier (BBB) leakage hypothesis (Fisher, 1979; Wardlaw et al., 2013b; Brown et al., 2018; Bown et al., 2022b). Vascular risk factors, such as hypertension, could lead to endothelial dysfunction potentially resulting in BBB leakage, rarefaction of adjacent white matter, pericyte loss, arteriolar thrombosis, microbleeds, and finally failure of ISF drainage.

Vascular disease can be caused by hypertension, as shown by early MRI and histopathology data (Braffman et al., 1988). This vascular maladaptation could stem from continued pulsatile barotrauma of affected blood vessels (Gutierrez et al., 2015). However, the association between hypertension and centrum semiovale MVPVS is ambiguous: whereas some studies suggest an increased MVPVS burden in hypertension (Hurford et al., 2014; Zhang et al., 2016; Arba et al., 2018; Lara et al., 2022), some studies do not confirm such a relation (Potter et al., 2015b; Riba-Llena et al., 2016) or show a decreased MVPVS burden in hypertension (Charidimou et al., 2014). In fact, accumulating evidence indicates that vascular risk factors such as hypertension are rather associated with basal ganglia MVPVS (Klarenbeek et al., 2013), which was corroborated by a recent meta-analysis (Francis et al., 2019). A potential explanation for this observation stems from imaging studies in hypertensive rats showing abnormal CSF flow dynamics and potentially perivascular clearance (Mortensen et al., 2019).

Hypertension is linked to reduced cerebrovascular reactivity (Hajjar et al., 2010). Cerebrovascular reactivity represents the dynamic ability of cerebral blood vessels to adjust cerebral blood flow in response to vasoactive stimuli (Liu et al., 2019). Reduced cerebrovascular reactivity has been associated with higher MVPVS burden (Blair et al., 2020; Kapoor et al., 2022) which has also been substantiated in rodents (Hadaczek et al., 2006; Kress et al., 2014). A 5-year prospective study employing flow MRI in 122 participants found that white matter lesions and MVPVS precede the increase in arterial pulsatility index, a measure of vascular resistance (Vikner et al., 2022). However, additional data is required to elucidate whether dilation of perivascular spaces are a consequence, cause, or bystander phenomenon to cerebrovascular reactivity deficits (Kapoor et al., 2022).

Whereas amplified vascular pulsations by hypertension could lead to vascular damage (and consequently impaired ISF drainage), reduced vascular pulsation could also lead to impaired ISF dynamics, as shown in rodent studies (Iliff et al., 2013). Interestingly, patients with carotid stenosis and thus potentially lower downstream vascular pulsations show higher basal ganglia MVPVS burden (Sahin et al., 2015) and diffusivity (Liu et al., 2021).

Intracerebral bleeding and stroke are ultimate consequences of vascular disease. Along these lines, intracerebral bleeding has been associated with MVPVS, as shown for symptomatic intracranial hemorrhage (Best et al., 2020), cerebral microbleeds (Wang et al., 2019), and CAA (Boulouis et al., 2017). In CAA, this only seems to apply to centrum semiovale MVPVS (Boulouis et al., 2017), which stands in contrast to basal ganglia MVPVS, as shown by florbetapir PET (Raposo et al., 2019). Thus, this pathomechanism seems to be more specific to CAA, while other factors might be relevant in hypertensive intracerebral hemorrhages (Tsai et al., 2021).

Additional pathomechanisms for the enlargement of PVS have been debated for ischemic pathology. Hemodynamically compromised individuals with atherosclerotic large vessel disease show higher levels of MVPVS (Mikami et al., 2018). Based on this observation, it has been hypothesized that MVPVS could serve as fluid absorbers in such a situation. In acute stroke, the data on MVPVS are less consistent: MVPVS seem to either vanish (Mikami et al., 2018) or increase (Zhang et al., 2016; Yu et al., 2022), possibly depending on the exact timing and/or extent of tissue damage. For example, in rodent stroke models, an early acute fluid inflow into perivascular spaces has been observed, which appears to drive formation of acute edema following ischemia (Mestre et al., 2020). In addition, both post-stroke (Wardlaw et al., 2009) and older age (Bake et al., 2009; Li et al., 2019) seem to be associated with BBB dysfunction, which is in turn associated with MVPVS. This suggests that MVPVS could indicate early BBB malfunction with abnormal ISF dynamics.

##### 3.3.1.3. Venous reflux and CSF-ISF efflux routes

Also, venous pathology has been associated with the enlargement of PVS: disruption of deep medullary veins was associated with increased burden of basal ganglia MVPVS (Zhang et al., 2022). Similarly, cerebral venous reflux after hypertensive intracerebral hemorrhages was associated with a larger number of basal ganglia MVPVS (Tsai et al., 2022).

It has also been speculated that the MVPVS enlargement in long-duration space travelers (Barisano et al., 2022a; Hupfeld et al., 2022) may be caused by venous pathology (Wostyn et al., 2022a,b). Concretely, a cephalad venous fluid shift would result in impaired cerebral venous outflow and thus reduced CSF/ISF dynamics. Consequently, the CSF may stagnate and accumulate at periarterial sites with dilation of the periarterial spaces. Along these lines, a brain upward shift could also obstruct major CSF-ISF efflux routes and/or dural lymphatics such as the superior sagittal sinus or bridging veins (Barisano et al., 2022a,b). Compromised CSF-ISF efflux by clogging of blood degradation might also cause MVPVS enlargement after subarachnoid hemorrhage (Kim J. et al., 2022).

##### 3.3.1.4. Sleep and time of day

Poorer sleep quality has been associated with increased MVPVS burden in a variety of diseases and also healthy adults (Berezuk et al., 2015; Baril et al., 2022; Del Brutto et al., 2022a; Wang et al., 2022). Although the pathomechanism behind this link is still under debate, sleep has been speculated to be a critical factor in CNS fluid dynamics (Brown et al., 2018). With this, poor sleep could contribute to less efficient ISF-CSF drainage (Baril et al., 2022). But also in individuals with stable sleep habits, MVPVS volumes can increase at later times of the day, possibly mediated by higher fluid amount within the MVPVS (Barisano et al., 2021).

#### 3.2.2. Hypothesis on spiral elongation of vessels/tortuous vessels

Although no direct evidence supports this hypothesis, it has been speculated that spiral elongation of arterial vessels could result in MVPVS (Wardlaw et al., 2013b; Ruchoux et al., 2021). Increasing space requirements and/or vascular pulsations of tortuous arteries could lead to dilation of perivascular spaces (Figure 3B). A similar mechanism has been proposed for MVPVS of the anterior temporal lobe: vascular tortuosity could lead to compression of small communicating fluid channels resulting in opercular perivascular cysts (Salzman et al., 2005; McArdle et al., 2020). Here, a tortuous middle cerebral artery branch could compress communicating fluid channels between the subarachnoid and perivascular spaces in the adjacent cortex.

It has also been suggested that ageing is associated with increased vascular tortuosity, which would further emphasize the link between aging and MVPVS (Spangler et al., 1994; Brown et al., 2002; Thore et al., 2007).

#### 3.3.3. Hypothesis on brain atrophy and perivascular myelin loss

MRI-visible perivascular spaces could be a sign of focal *ex vacuo* atrophy and/or demyelination of adjacent brain tissue (Groeschel et al., 2006; Wardlaw et al., 2013b). Tissue loss surrounding the perivascular compartment would result in secondary dilation of perivascular spaces (Figure 3C). If this were the case, one could expect that higher MVPVS burden would be associated with lower brain volume measures. Such an association has mostly been studied in stroke, Alzheimer’s dementia, multiple sclerosis, and healthy individuals, yet the data across these studies is inconsistent. Whereas some publications report a negative correlation between MVPVS and various brain volume measures, e.g., in stroke (Potter et al., 2015b; Wang et al., 2016; Zhang et al., 2016), Alzheimer’s disease (Gertje et al., 2021), MS (Kilsdonk et al., 2015; Liu et al., 2022), NMOSD (Cacciaguerra et al., 2022), or health (Chen et al., 2011; Sim et al., 2020), several studies do not confirm such an association (stroke (Arba et al., 2018), Alzheimer’s disease (Chen et al., 2011), MS (Wuerfel et al., 2008; Conforti et al., 2014, 2016; Favaretto et al., 2017; Cavallari et al., 2018; Wooliscroft et al., 2020; Kolbe et al., 2022), and health (Huang et al., 2021)).

**FIGURE 4:**
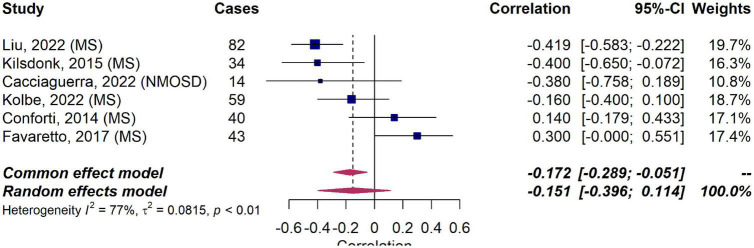
Forest plot of correlation coefficients between MRI-visible perivascular spaces (MVPVS) and brain volume measures. Pooled analyses of studies comparing the correlation between MVPVS and brain volume measures shows no statistically significant correlation (a negative correlation indicates that higher MVPVS levels correlate with lower brain volume measures). Correlation coefficients were extracted and pooled using the random effects DerSimonian-Laird method. CI, confidence interval; MS, multiple sclerosis; NMOSD, neuromyelitis optica spectrum disorder.

Based on this inconsistent data, we meta-analyzed correlation in six studies in MS (Conforti et al., 2014; Kilsdonk et al., 2015; Favaretto et al., 2017; Kolbe et al., 2022; Liu et al., 2022) and NMOSD (Cacciaguerra et al., 2022) reporting correlation coefficients between MVPVS and brain volume measures (including a total of 258 MS patients and 14 NMOSD patients). The employed MRI parameters for the included studies are summarized in Table 1 (the remaining studies could not be included, either due to missing quantitative data or an insufficient number of publications per disease). In this meta-analysis, we did not identify a significant (negative) correlation between MVPVS and brain volume [R: −0.15 (95%-CI −0.40–0.11)] (Figure 4). However, there was substantial heterogeneity between studies assessing whole brain MVPVS (*I*^2^ = 77%) (Higgins and Thompson, 2002). Of note, age was not a significant moderator for MVPVS in the meta-analysis (*p* = 0.28) (age range between 35 and 49). The effect size did not change in a meta-analysis only comprising studies in MS [*R* = −0.12 (95%-CI −0.40–0.17)] or when applying the Knapp-Hartung method for the meta-analysis.

**TABLE 1:** Employed magnetic resonance imaging (MRI) parameters of studies included in the meta-analysis.

Study	B_0_ magnetic field strength	Sequences to assess MVPVS	MVPVS assessment method	Image resolution
Cacciaguerra et al., 2022 (NMOSD)	3T	3D T2 w	Potter scale (Potter et al., 2015a) (semiquantitative)	1 mm isotropic
Conforti et al., 2014 (MS)	3T	2D T2 w-PD fast spin echo, 2D T2 w-FLAIR, 3D T1 w spoiled gradient-recalled	manual/semi-automatic assessment using MIPAV (MVPVS number, area, and volume)	T1 w: 1 × 1.2 × 1.2 mm
Favaretto et al., 2017 (MS)	3T	3D T1 w, 3D T2 w-FLAIR, 2D phase sensitive inversion recovery	Manual count/segmentation using ITK-SNAP (MVPVS number and volume)	T1 w and T2 w-FLAIR: 1 mm isotropic, 2D PSIR: 1 × 1 × 3 mm
Kilsdonk et al., 2015 (MS)	7T	3D T1 w	Manual count in 5 brain regions using MIPAV (MVPVS number)	0.8 mm isotropic (nominal)
Kolbe et al., 2022 (MS)	3T	2D T2 w fast spin echo	Potter scale (Potter et al., 2015a) (semiquantitative)	0.8 × 0.8 × 5 mm
Liu et al., 2022 (MS)	3T	T2 w	Potter scale (Potter et al., 2015a) (semiquantitative)	Not reported

MVPVS, MRI-visible perivascular spaces; FLAIR, fluid-attenuated inversion recovery; MS, multiple sclerosis; NMOSD, neuromyelitis optica spectrum disorder; PD, proton density; PSIR, phase sensitive inversion recovery.

#### 3.3.4. Hypothesis on immune cell accumulation in the perivascular space

A relatively small longitudinal study in MS patients has speculated that MVPVS dilation could represent perivascular accumulation of immune cells prior to a neuroinflammatory insult which could result in enlargement of the perivascular space (Wuerfel et al., 2008; Figure 3D). This notion was based on data showing higher MVPVS volumes in MS patients with gadolinium-enhancing MRI lesions. Although MS lesions form in a perivenular configuration that can be imaged via specific types of susceptibility-sensitive MRI (Sati et al., 2016), findings showing perivascular cell accumulation in MVPVS in MS have not been reproduced to date (Achiron and Faibel, 2002; Cavallari et al., 2018; Kolbe et al., 2022) [reviewed in Granberg et al. (2020)].

### 3.4. Temporal evolution of MVPVS

#### 3.4.1. Neuroinflammatory diseases

Two studies investigated the temporal dynamics of MVPVS in MS. One study had a subset of 18 MS patients who were prospectively followed monthly for 1 year (Wuerfel et al., 2008). These MS patients showed higher MVPVS volumes in MRI scans positive for contrast-enhancing lesions compared to scans without such lesions. MVPVS volumes did not decrease in patients who shifted from a state with to a state without contrast-enhancing lesions. The other study comprised 59 MS patients with a mean follow-up time of 20 months (Kolbe et al., 2022). Across the cohort, the number of MVPVS increased in the centrum semiovale (+4.1 MVPVS per year) but not in the basal ganglia or midbrain, and MVPVS increase in the midbrain was associated with white matter lesion volume change. Higher MVPVS numbers were not associated with prospective brain volume loss, and MVPVS change was not associated with contrast-enhancing lesions, brain volume change, or physical disability of MS patients.

MRI-visible perivascular spaces changes have also been studied in primary angiitis of the central nervous system (PACNS) (Campi et al., 2001). In a small study in patients with contrast-enhancing parenchymal lesions, 4/6 patients showed MVPVS with concomitant contrast enhancement which subsequently regressed during the follow-up (12–60 months).

#### 3.4.2. Vascular diseases

In a cohort with hypertension, 23 of 233 individuals (10%) showed increasing numbers of MVPVS in the basal ganglia over a 4-year follow-up period (Jiménez-Balado et al., 2020). Similar rates of basal ganglia MVPVS progression have been observed after subarachnoid hemorrhage (6%) (Kim J. et al., 2022). Interestingly, the MVPVS progression rates were much higher in the centrum semiovale after subarachnoid hemorrhage (31%).

#### 3.4.3. Metabolic disorders

Two studies investigated MVPVS changes in mucopolysaccharidoses. The initial study found that corpus callosum MVPVS increased in 3 of 18 patients, decreased in 2 patients, and remained stable in the remaining 13 patients during a follow-up period of 37 months (Manara et al., 2011). MVPVS in CNS locations outside the corpus callosum were stable in most patients (17/19 patients, 89%). Similar MVPVS dynamics were reported in the other study (Alqahtani et al., 2014).

#### 3.4.4. Tumefactive MVPVS and opercular perivascular cysts

In contrast to MVPVS, which can be observed in healthy individuals of all age groups, opercular perivascular cysts (type IV MVPVS) are much rarer (Lim et al., 2015). This MVPVS subtype mostly occurs in the anterior temporal lobe and less commonly in the frontal operculum and can be associated with perilesional T2 signal (McArdle et al., 2020). A recent case series reported on 18 individuals with opercular perivascular cysts (McArdle et al., 2020): of 13 subjects with follow-up ranging from 2 months to approximately 10 years, 11 exhibited stable MVPVS dimensions while 1 showed a slight increase in MVPVS volume within a 7-month period.

Very large MVPVS, referred to as giant or tumefactive MVPVS, can also occur in other regions of the CNS, including the basal ganglia (Tsutsumi et al., 2011), the centrum semiovale (Taniguchi et al., 2017), the hippocampus (Rivet et al., 2017), the corpus callosum (Manara et al., 2010), and the midbrain (Rocha et al., 2013). Several case reports have confirmed the mostly stable nature of these structures (Ogawa et al., 1995; Sawada et al., 1999; Flors et al., 2010; Tsutsumi et al., 2011; Sankararaman et al., 2013; Tseng et al., 2013; Taniguchi et al., 2017), some of them with follow-up periods of up to 7 years (Manara et al., 2010). However, a subset of these MVPVS can enlarge over time (Mehta et al., 2013; Gopinath et al., 2018; Yamaguchi et al., 2021). This can result in compression of adjacent CNS structures with concomitant neurological disorders such as non-communicating hydrocephalus (Papayannis et al., 2003), necessitating surgical decompression (Rocha et al., 2013; Rivet et al., 2017). A systematic review summarized MRI findings from 164 cases with tumefactive MVPVS (Kwee and Kwee, 2019): whereas most tumefactive basal ganglia MVPVS remained stable (23/24 cases), 7 of 64 participants (11%) showed enlargement of MVPVS in the pontomesencephalic region.

#### 3.4.5. Healthy individuals

A recent study assessed MVPVS progression in the Atahualpa project cohort, which comprises healthy community-dwelling elderly Ecuadorian (mean age: 66). Within more than 1,700 patient-years and a mean follow-up of 6.5 years, 56 of 263 individuals (21%) showed mostly mild MVPVS progression (Del Brutto et al., 2022b). Another community-based study followed 191 older subjects (mean age: 68) over 7 years; 65 of the study participants (34%) showed MVPVS progression during the follow-up time (Xia et al., 2020).

## 4. Discussion

### 4.1. Main findings

This study aimed at systematically summarizing potential etiologies of MVPVS as well as their temporal dynamics. Based on a comprehensive literature search, we identified four major, partly overlapping hypothesized etiologies of MVPVS (Figure 4): (1) Impairment of ISF circulation, potentially driven by deposition of protein aggregates, arterial/venous pathology, sleep disturbances, and disruption of CSF-ISF efflux routes; (2) Spiral elongation of arteries; (3) Brain atrophy and/or perivascular myelin loss; and (4) Immune cell accumulation in the perivascular space. Overall, only a few studies have investigated temporal dynamics of MVPVS. These studies mainly show that MVPVS present with limited evolution over time.

### 4.2. Findings in the context of existing evidence

Although these four proposed etiologies constitute different pathomechanisms for MVPVS, they are partly overlapping. For example, chronic hypertension can cause both BBB damage (Ungvari et al., 2021) and vascular tortuosity (Ciuricã et al., 2019), and hypertension can also be associated with brain atrophy (Wiseman et al., 2004). Nevertheless, it is likely that these etiologies contribute differently to MVPVS emergence and/or dynamics in different neurological entities. For example, in vascular diseases such as small vessel disease and stroke, impaired dynamics of ISF could be a main driver of MVPVS formation, e.g., via endothelial dysfunction, vessel-wall thickening, luminal occlusion, and BBB leakage (Wardlaw et al., 2013b). In MS or other neuroinflammatory diseases, immune cell accumulation could be a possible etiology (Wuerfel et al., 2008), yet this is a highly controversial hypothesis for which there is insufficient evidence to date (Granberg et al., 2020).

In aging, which has consistently been associated with increased MVPVS burden (Zong et al., 2020; Barisano et al., 2021; Kim H. G. et al., 2022; Lara et al., 2022), *ex vacuo* brain atrophy (Wardlaw et al., 2020), and vascular tortuosity (Spangler et al., 1994; Brown et al., 2002; Thore et al., 2007) could be more prominent drivers for MVPVS emergence. In addition, increasing exposure to vascular risk factors could also contribute to enlargement of MVPVS in aging (Lara et al., 2022). Of note, however, in our meta-analysis, there was no significant correlation between MVPVS and brain volumes.

MRI-visible perivascular spaces are also a common neuroimaging finding in younger (Piantino et al., 2020; Zou et al., 2022) and healthy individuals (Del Brutto et al., 2022b; Kim H. G. et al., 2022). In this population, tortuosity of arterial blood vessels could be an etiology for MVPVS. Furthermore, a heritable component of MVPVS burden has been suggested (Luo et al., 2017; Barisano et al., 2021) [reviewed in Bown et al. (2022a)].

It is generally assumed that MVPVS mostly adjoin arterial vessels (Ineichen et al., 2022), which is bolstered by small imaging studies (Bouvy et al., 2014; George et al., 2021). However, structural differences between periarterial and perivenous spaces are scarcely investigated, and it thus remains unclear to what degree these presumable etiologies contribute to MVPVS emergence within arteries or veins. It can be speculated that arterial alterations, such as hypertension or vascular tortuosity would contribute to the enlargement of periarterial spaces only, whereas e*x vacuo* atrophy would contribute to enlargement at both sites. Along these lines, different mechanisms could contribute to MVPVS emergence in the basal ganglia or the supratentorial white matter, but again, structural MVPVS differences between these two anatomical regions are insufficiently understood. Additionally, it has been suggested that basal ganglia MVPVS are associated with vascular pathology such as hypertension (Klarenbeek et al., 2013; Francis et al., 2019) or venous reflux (Tsai et al., 2022; Zhang et al., 2022), whereas centrum semiovale MVPVS are associated with amyloid angiopathy (Boulouis et al., 2017).

Regarding MVPVS evolution over time, only a few and mostly small studies assessed temporal dynamics of MVPVS. Based on these limited data, it seems that number of MVPVS are for the most part stable over relatively long observation periods (median follow-up time of eligible studies was 36 months). However, in a minority of cases, they might increase in number and size with aging. Additional mechanisms could contribute to MVPVS dynamics in neuroinflammatory diseases such as PACNS (Campi et al., 2001).

### 4.3. Limitations

Our study has some limitations: First, a wide variety of imaging methods and MVPVS evaluation methods have been employed to assess MVPVS. Even within studies, the interrater agreement for MVPVS detection can be moderate to substantial (Cohen’s kappa: 0:58–0.91) (Wang et al., 2019; Best et al., 2020; Javierre-Petit et al., 2020; Ciampa et al., 2021; Song et al., 2021). This heterogeneity could have biased our narrative summary. Second, for assessing the correlation between MVPVS and brain atrophy, we pooled studies with various methodological backgrounds for summary estimates (Table 1), and the meta-analysis could only be conducted for a subgroup of patients with neuroinflammatory diseases. Nonetheless, we mitigated this partly by only including studies that reported correlation coefficients, i.e., uniform outcome measures, and by applying a random effect model meta-analysis. Third, it is noteworthy that only very few studies (Van Veluw et al., 2016) provided pathology data for assessing MVPVS etiopathogenesis, and the hypotheses presented even in these studies were mostly based on imaging data, which could also bias the conclusions drawn. Fourth, only a few animal studies assessing etiopathogenesis of enlarged perivascular spaces were eligible. With this, the proposed MVPVS etiopathogeneses remain speculative and further data is warranted to corroborate their validity.

## 5. Conclusion

Our study summarizes potential etiologies (Figure 4) and temporal dynamics of MVPVS. Although a variety of etiologies have been proposed, they are only partly supported by actual data. Thus, advanced MRI methods, e.g., to monitor fluid dynamics within perivascular spaces, as well as ultra-high-field MRI to gain high-resolution insights into perivascular spaces, could give more detailed understanding of MVPVS etiopathogenesis (Ineichen et al., 2022). In addition, larger studies with longer follow-up times and harmonized MRI across sites investigating temporal MVPVS dynamics are warranted, preferentially boosted by automated and thus less biased detection of MVPVS. Finally, correlating MVPVS with their corresponding histopathology could give key insights into their pathophysiology.

## Data availability statement

The original contributions presented in this study are included in the article/supplementary material, further inquiries can be directed to the corresponding author.

## Author contributions

SO and BI: abstract and full text screening as well as data extraction and writing the initial manuscript draft. All authors contributed to the study conception and critical revision of the manuscript.

## Footnotes

1
https://www.crd.york.ac.uk/PROSPERO/

## References

[B1] Achiron A. Faibel M. (2002). Sandlike appearance of Virchow-Robin spaces in early multiple sclerosis: A novel neuroradiologic marker. *Am. J. Neuroradiol.* 23 376–380. 11901003 PMC7975312

[B2] Alqahtani E. Huisman T. A. Boltshauser E. Scheer I. Güngör T. Tekes A. (2014). Mucopolysaccharidoses type I and II: New neuroimaging findings in the cerebellum. *Eur. J. Paediatr. Neurol.* 18 211–217. 10.1016/j.ejpn.2013.11.014 24423630

[B3] Arba F. Quinn T. J. Hankey G. J. Lees K. R. Wardlaw J. M. Ali M. (2018). Enlarged perivascular spaces and cognitive impairment after stroke and transient ischemic attack. *Int. J. Stroke* 13 47–56. 10.1177/1747493016666091 27543501

[B4] Bake S. Friedman J. A. Sohrabji F. (2009). Reproductive age-related changes in the blood brain barrier: Expression of IgG and tight junction proteins. *Microvasc. Res.* 78 413–424. 10.1016/j.mvr.2009.06.009 19591848 PMC2784250

[B5] Ballerini L. Lovreglio R. Valdes Hernandez M. D. C. Ramirez J. MacIntosh B. J. Black S. E. (2018). Perivascular spaces segmentation in brain MRI using optimal 3D filtering. *Sci. Rep.* 8:2132. 10.1038/s41598-018-19781-5 29391404 PMC5794857

[B6] Banerjee G. Kim H. J. Fox Z. Jager H. R. Wilson D. Charidimou A. (2017). MRI-visible perivascular space location is associated with Alzheimer’s disease independently of amyloid burden. *Brain* 140 1107–1116. 10.1093/brain/awx003 28335021

[B7] Baril A. A. Pinheiro A. A. Himali J. J. Beiser A. Sanchez E. Pase M. P. (2022). Lighter sleep is associated with higher enlarged perivascular spaces burden in middle-aged and elderly individuals. *Sleep Med.* 100 558–564. 10.1016/j.sleep.2022.10.006 36308914 PMC9815141

[B8] Barisano G. Sepehrband F. Collins H. R. Jillings S. Jeurissen B. Taylor J. A. (2022a). The effect of prolonged spaceflight on cerebrospinal fluid and perivascular spaces of astronauts and cosmonauts. *Proc. Natl. Acad. Sci. U.S.A.* 119:e2120439119. 10.1073/pnas.2120439119 PMC9169932 35412862

[B9] Barisano G. Tomilovskaya E. Roberts D. R. Wuyts F. L. (2022b). Reply to Wostyn et al. Potential models for perivascular space (PVS) enlargement and spaceflight-associated neuro-ocular syndrome (SANS). *Proc. Natl. Acad. Sci. U.S.A.* 119:e2208241119. 10.1073/pnas.2208241119 35858379 PMC9371741

[B10] Barisano G. Sheikh-Bahaei N. Law M. Toga A. W. Sepehrband F. (2021). Body mass index, time of day and genetics affect perivascular spaces in the white matter. *J. Cereb. blood Flow Metab.* 41 1563–1578. 10.1177/0271678X20972856 33183133 PMC8221772

[B11] Berezuk C. Ramirez J. Gao F. Scott C. J. Huroy M. Swartz R. H. (2015). Virchow-Robin spaces: Correlations with polysomnography-derived sleep parameters. *Sleep* 38 853–858. 10.5665/sleep.4726 26163465 PMC4434551

[B12] Best J. G. Barbato C. Ambler G. Du H. Banerjee G. Wilson D. (2020). Association of enlarged perivascular spaces and anticoagulant-related intracranial hemorrhage. *Neurology* 95 e2192–e2199. 10.1212/WNL.0000000000010788 32934168 PMC7713790

[B13] Blair G. W. Thrippleton M. J. Shi Y. Hamilton I. Stringer M. Chappell F. (2020). Intracranial hemodynamic relationships in patients with cerebral small vessel disease. *Neurology* 94 e2258–e2269. 32366534 10.1212/WNL.0000000000009483 PMC7357294

[B14] Boespflug E. L. Schwartz D. L. Lahna D. Pollock J. Iliff J. J. Kaye J. A. (2018). MR imaging-based multimodal autoidentification of perivascular spaces (mMAPS): Automated morphologic segmentation of enlarged perivascular spaces at clinical field strength. *Radiology* 286 632–642. 10.1148/radiol.2017170205 28853674 PMC5790307

[B15] Boulouis G. Charidimou A. Pasi M. Roongpiboonsopit D. Xiong L. Auriel E. (2017). Hemorrhage recurrence risk factors in cerebral amyloid angiopathy: Comparative analysis of the overall small vessel disease severity score versus individual neuroimaging markers. *J. Neurol. Sci.* 380 64–67. 10.1016/j.jns.2017.07.015 28870591 PMC5678990

[B16] Boutinaud P. Tsuchida A. Laurent A. Adonias F. Hanifehlou Z. Nozais V. (2021). 3D segmentation of perivascular spaces on T1-weighted 3 Tesla MR images with a convolutional autoencoder and a U-shaped neural network. *Front. Neuroinform.* 15:641600. 10.3389/fninf.2021.641600 34262443 PMC8273917

[B17] Bouvy W. H. Biessels G. J. Kuijf H. J. Kappelle L. J. Luijten P. R. Zwanenburg J. J. (2014). Visualization of perivascular spaces and perforating arteries with 7 T magnetic resonance imaging. *Invest. Radiol.* 49 307–313. 10.1097/RLI.0000000000000027 24473365

[B18] Bown C. W. Khan O. A. Liu D. Remedios S. W. Pechman K. R. Terry J. G. (2022b). Enlarged perivascular space burden associations with arterial stiffness and cognition. *Neurobiol. Aging.* 10.1016/j.neurobiolaging.2022.10.014 36446680 PMC9957942

[B19] Bown C. W. Carare R. O. Schrag M. S. Jefferson A. L. (2022a). Physiology and clinical relevance of enlarged perivascular spaces in the aging brain. *Neurology* 98 107–117. 10.1212/WNL.0000000000013077 34810243 PMC8792814

[B20] Braffman B. H. Zimmerman R. A. Trojanowski J. Q. Gonatas N. K. Hickey W. F. Schlaepfer W. W. (1988). Brain MR: Pathologic correlation with gross and histopathology. 1. Lacunar infarction and Virchow-Robin spaces. *Am. J. Roentgenol.* 151 551–558. 10.2214/ajr.151.3.551 3261517

[B21] Brown R. Benveniste H. Black S. E. Charpak S. Dichgans M. Joutel A. (2018). Understanding the role of the perivascular space in cerebral small vessel disease. *Cardiovasc. Res.* 114 1462–1473. 29726891 10.1093/cvr/cvy113 PMC6455920

[B22] Brown W. R. Moody D. M. Challa V. R. Thore C. R. Anstrom J. A. (2002). Venous collagenosis and arteriolar tortuosity in leukoaraiosis. *J. Neurol. Sci.* 203–204 159–163. 10.1016/s0022-510x(02)00283-6 12417376

[B23] Cacciaguerra L. Carotenuto A. Pagani E. Mistri D. Radaelli M. Martinelli V. (2022). Magnetic resonance imaging evaluation of perivascular space abnormalities in neuromyelitis optica. *Ann. Neurol.* 92 173–183. 10.1002/ana.26419 35596582 PMC9544484

[B24] Campi A. Benndorf G. Filippi M. Reganati P. Martinelli V. Terreni M. R. (2001). Primary angiitis of the central nervous system: Serial MRI of brain and spinal cord. *Neuroradiology* 43 599–607. 11548164 10.1007/s002340100561

[B25] Cavallari M. Egorova S. Healy B. C. Palotai M. Prieto J. C. Polgar-Turcsanyi M. (2018). Evaluating the association between enlarged perivascular spaces and disease worsening in multiple sclerosis. *J. Neuroimaging* 28 273–277. 10.1111/jon.12490 29226505

[B26] Charidimou A. Boulouis G. Pasi M. Auriel E. van Etten E. S. Haley K. (2017). MRI-visible perivascular spaces in cerebral amyloid angiopathy and hypertensive arteriopathy. *Neurology* 88 1157–1164. 28228568 10.1212/WNL.0000000000003746 PMC5373782

[B27] Charidimou A. Jäger R. H. Peeters A. Vandermeeren Y. Laloux P. Baron J.-C. (2014). White matter perivascular spaces are related to cortical superficial siderosis in cerebral amyloid angiopathy. *Stroke* 45 2930–2935. 25116879 10.1161/STROKEAHA.114.005568

[B28] Charidimou A. Meegahage R. Fox Z. Peeters A. Vandermeeren Y. Laloux P. (2013). Enlarged perivascular spaces as a marker of underlying arteriopathy in intracerebral haemorrhage: A multicentre MRI cohort study. *J. Neurol. Neurosurg. Psychiatry* 84 624–629. 10.1136/jnnp-2012-304434 23412074 PMC3905629

[B29] Chen W. Song X. Zhang Y. (2011). Assessment of the Virchow-Robin Spaces in Alzheimer disease, mild cognitive impairment, and normal aging, using high-field MR imaging. *Am. J. Neuroradiol.* 32 1490–1495. 10.3174/ajnr.A2541 21757525 PMC7964361

[B30] Ciampa I. Operto G. Falcon C. Minguillon C. Castro de Moura M. Piñeyro D. (2021). Genetic predisposition to Alzheimer’s disease is associated with enlargement of perivascular spaces in centrum semiovale region. *Genes* 12:825. 10.3390/genes12060825 34072165 PMC8226614

[B31] Ciuricã S. Lopez-Sublet M. Loeys B. L. Radhouani I. Natarajan N. Vikkula M. (2019). Arterial tortuosity: Novel implications for an old phenotype. *Hypertension* 73 951–960. 30852920 10.1161/HYPERTENSIONAHA.118.11647

[B32] Cochran W. G. (1954). The combination of estimates from different experiments. *Biometrics* 10 101–129.

[B33] Conforti R. Cirillo M. Sardaro A. Caiazzo G. Negro A. Paccone A. (2016). Dilated perivascular spaces and fatigue: Is there a link? Magnetic resonance retrospective 3Tesla study. *Neuroradiology* 58 859–866. 10.1007/s00234-016-1711-0 27423658

[B34] Conforti R. Cirillo M. Saturnino P. P. Gallo A. Sacco R. Negro A. (2014). Dilated Virchow-Robin spaces and multiple sclerosis: 3 T magnetic resonance study. *Radiol. Med.* 119 408–414. 24297592 10.1007/s11547-013-0357-9

[B35] Del Brutto O. H. Mera R. M. Costa A. F. Rumbea D. A. Recalde B. Y. Castillo P. R. (2022a). Long coronavirus disease-related persistent poor sleep quality and progression of enlarged perivascular spaces. A longitudinal study. *Sleep* 45:zsac168. 10.1093/sleep/zsac168 35878737 PMC9384510

[B36] Del Brutto O. H. Mera R. M. Costa A. F. Rumbea D. A. Recalde B. Y. Del Brutto V. J. (2022b). Patterns of progression of cerebral small vessel disease markers in older adults of Amerindian ancestry: A population-based, longitudinal prospective cohort study. *Aging Clin. Exp. Res.* 34 2751–2759. 10.1007/s40520-022-02223-8 35999426 PMC9398047

[B37] DerSimonian R. Laird N. (1986). Meta-analysis in clinical trials. *Control. Clin. Trials* 7 177–188. 3802833 10.1016/0197-2456(86)90046-2

[B38] Favaretto A. Lazzarotto A. Riccardi A. Pravato S. Margoni M. Causin F. (2017). Enlarged Virchow Robin spaces associate with cognitive decline in multiple sclerosis. *PLoS One* 12:e0185626. 10.1371/journal.pone.0185626 29045421 PMC5646763

[B39] Fisher C. M. (1979). Capsular infarcts: The underlying vascular lesions. *Arch. Neurol.* 36 65–73. 420625 10.1001/archneur.1979.00500380035003

[B40] Flors L. Leiva-Salinas C. Cabrera G. Mazón M. Poyatos C. (2010). Obstructive hydrocephalus due to cavernous dilation of Virchow-Robin spaces. *Neurology* 74:1746. 10.1212/WNL.0b013e3181e04312 20498444

[B41] Francis F. Ballerini L. Wardlaw J. M. (2019). Perivascular spaces and their associations with risk factors, clinical disorders and neuroimaging features: A systematic review and meta-analysis. *Int. J. Stroke* 14 359–371. 10.1177/1747493019830321 30762496

[B42] George I. Arrighi-Allisan A. Delman B. Balchandani P. Horng S. Feldman R. (2021). A novel method to measure venular perivascular spaces in patients with MS on 7T MRI. *Am. J. Neuroradiol.* 42 1069–1072. 10.3174/ajnr.A7144 33858821 PMC8191677

[B43] Gertje E. C. van Westen D. Panizo C. Mattsson-Carlgren N. Hansson O. (2021). Association of enlarged perivascular spaces and measures of small vessel and Alzheimer disease. *Neurology* 96 e193–e202. 33046608 10.1212/WNL.0000000000011046

[B44] Gopinath M. Nagesh C. Kesavadas C. (2018). Post radiation evolution of giant Virchow-Robin spaces in a case of pituitary macroadenoma. *Indian J. Radiol. Imaging* 28 373–374. 10.4103/ijri.IJRI_335_17 30319219 PMC6176662

[B45] Granberg T. Moridi T. Brand J. S. Neumann S. Hlavica M. Piehl F. (2020). Enlarged perivascular spaces in multiple sclerosis on magnetic resonance imaging: A systematic review and meta-analysis. *J. Neurol.* 267 3199–3212. 10.1007/s00415-020-09971-5 32535680 PMC7577911

[B46] Groeschel S. Chong W. K. Surtees R. Hanefeld F. (2006). Virchow-Robin spaces on magnetic resonance images: Normative data, their dilatation, and a review of the literature. *Neuroradiology* 48 745–754. 10.1007/s00234-006-0112-1 16896908

[B47] Gutierrez J. Elkind M. S. Cheung K. Rundek T. Sacco R. L. Wright C. B. (2015). Pulsatile and steady components of blood pressure and subclinical cerebrovascular disease: The Northern Manhattan Study. *J. Hypertens.* 33:2115. 10.1097/HJH.0000000000000686 26259124 PMC4871260

[B48] Hadaczek P. Yamashita Y. Mirek H. Tamas L. Bohn M. C. Noble C. (2006). The “perivascular pump” driven by arterial pulsation is a powerful mechanism for the distribution of therapeutic molecules within the brain. *Mol. Ther.* 14 69–78. 10.1016/j.ymthe.2006.02.018 16650807 PMC2730223

[B49] Hajjar I. Zhao P. Alsop D. Novak V. (2010). Hypertension and cerebral vasoreactivity: A continuous arterial spin labeling magnetic resonance imaging study. *Hypertension* 56 859–864. 10.1161/HYPERTENSIONAHA.110.160002 20876450 PMC3040032

[B50] Higgins J. P. Thompson S. G. (2002). Quantifying heterogeneity in a meta-analysis. *Stat. Med.* 21 1539–1558. 12111919 10.1002/sim.1186

[B51] Hou Y. Park S. H. Wang Q. Zhang J. Zong X. Lin W. (2017). Enhancement of perivascular spaces in 7 T MR image using Haar transform of non-local cubes and block-matching filtering. *Sci. Rep.* 7 1–12. 10.1038/s41598-017-09336-5 28819140 PMC5561084

[B52] Huang P. Zhu Z. Zhang R. Wu X. Jiaerken Y. Wang S. (2021). Factors associated with the dilation of perivascular space in healthy elderly subjects. *Front. Aging Neurosci.* 13:624732. 10.3389/fnagi.2021.624732 33841126 PMC8032856

[B53] Hupfeld K. E. Richmond S. B. McGregor H. R. Schwartz D. L. Luther M. N. Beltran N. E. (2022). Longitudinal MRI-visible perivascular space (PVS) changes with long-duration spaceflight. *Sci. Rep.* 12:7238. 10.1038/s41598-022-11593-y 35513698 PMC9072425

[B54] Hurford R. Charidimou A. Fox Z. Cipolotti L. Jager R. Werring D. J. (2014). MRI-visible perivascular spaces: Relationship to cognition and small vessel disease MRI markers in ischaemic stroke and TIA. *J. Neurol. Neurosurg. Psychiatry* 85 522–525. 10.1136/jnnp-2013-305815 24249785 PMC3995332

[B55] Iliff J. J. Wang M. Zeppenfeld D. M. Venkataraman A. Plog B. A. Liao Y. (2013). Cerebral arterial pulsation drives paravascular CSF-interstitial fluid exchange in the murine brain. *J. Neurosci.* 33 18190–18199. 10.1523/JNEUROSCI.1592-13.2013 24227727 PMC3866416

[B56] Ineichen B. V. Okar S. V. Proulx S. T. Engelhardt B. Lassmann H. Reich D. S. (2022). Perivascular spaces and their role in neuroinflammation. *Neuron* 110 3566–3581. 36327898 10.1016/j.neuron.2022.10.024 PMC9905791

[B57] Javierre-Petit C. Schneider J. A. Kapasi A. Makkinejad N. Tamhane A. A. Leurgans S. E. (2020). Neuropathologic and cognitive correlates of enlarged perivascular spaces in a community-based cohort of older adults. *Stroke* 51 2825–2833. 10.1161/STROKEAHA.120.029388 32757750 PMC7484322

[B58] Jeong S. H. Cha J. Park M. Jung J. H. Ye B. S. Sohn Y. H. (2022). Association of enlarged perivascular spaces with amyloid burden and cognitive decline in Alzheimer disease continuum. *Neurology.* 10.1212/WNL.0000000000200989 35985826

[B59] Jiménez-Balado J. Riba-Llena I. Maisterra O. Pizarro J. Palasí A. Pujadas F. (2020). Ambulatory blood pressure levels in the prediction of progression of cerebral small vessel disease. *J. Am. Geriatr. Soc.* 68 2232–2239. 10.1111/jgs.16568 32511756

[B60] Kapoor A. Yew B. Jang J. Y. Dutt S. Li Y. Alitin J. P. M. (2022). Older adults with perivascular spaces exhibit cerebrovascular reactivity deficits. *Neuroimage* 264:119746. 10.1016/j.neuroimage.2022.119746 36370956 PMC10033456

[B61] Kilsdonk I. D. Steenwijk M. D. Pouwels P. J. Zwanenburg J. J. Visser F. Luijten P. R. (2015). Perivascular spaces in MS patients at 7 Tesla MRI: A marker of neurodegeneration? *Mult. Scler.* 21 155–162. 10.1177/1352458514540358 25013150

[B62] Kim H. G. Shin N. Y. Nam Y. Yun E. Yoon U. Lee H. S. (2022). MRI-visible dilated perivascular space in the brain by age: The human connectome project. *Radiology* 306:213254. 10.1148/radiol.213254 36378031

[B63] Kim J. Joo B. Kim J. W. Park M. Ahn S. J. Park S. K. (2022). Aggravation of enlarged perivascular spaces in the centrum semiovale of patients with aneurysmal subarachnoid hemorrhage. *Clin. Neuroradiol.* 32 79–87. 10.1007/s00062-021-01098-y 34618170

[B64] Klarenbeek P. van Oostenbrugge R. J. Rouhl R. P. Knottnerus I. L. Staals J. (2013). Ambulatory blood pressure in patients with lacunar stroke: Association with total MRI burden of cerebral small vessel disease. *Stroke* 44 2995–2999. 23982717 10.1161/STROKEAHA.113.002545

[B65] Kolbe S. Garcia L. Yu N. Boonstra F. Clough M. Sinclair B. (2022). Lesion volume in relapsing multiple sclerosis is associated with perivascular space enlargement at the level of the basal ganglia. *Am. J. Neuroradiol.* 43 238–244. 10.3174/ajnr.A7398 35121585 PMC8985682

[B66] Kress B. T. Iliff J. J. Xia M. Wang M. Wei H. S. Zeppenfeld D. (2014). Impairment of paravascular clearance pathways in the aging brain. *Ann. Neurol.* 76 845–861. 25204284 10.1002/ana.24271 PMC4245362

[B67] Kwee R. M. Kwee T. C. (2007). Virchow-Robin spaces at MR imaging. *Radiographics* 27 1071–1086. 17620468 10.1148/rg.274065722

[B68] Kwee R. M. Kwee T. C. (2019). Tumefactive Virchow-Robin spaces. *Eur. J. Radiol.* 111 21–33. 30691661 10.1016/j.ejrad.2018.12.011

[B69] Lara F. R. Scruton A. L. Pinheiro A. Demissie S. Parva P. Charidimou A. (2022). Aging, prevalence and risk factors of MRI-visible enlarged perivascular spaces. *Aging* 14 6844–6858. 10.18632/aging.204181 35852852 PMC9512514

[B70] Li Y. Li M. Yang L. Qin W. Yang S. Yuan J. (2019). The relationship between blood-brain barrier permeability and enlarged perivascular spaces: A cross-sectional study. *Clin. Interv. Aging* 14 871–878. 31190773 10.2147/CIA.S204269 PMC6519012

[B71] Lim A. T. Chandra R. V. Trost N. M. McKelvie P. A. Stuckey S. L. (2015). Large anterior temporal Virchow-Robin spaces: Unique MR imaging features. *Neuroradiology* 57 491–499. 25614333 10.1007/s00234-015-1491-y

[B72] Liu H. Yang S. He W. Liu X. Sun S. Wang S. (2021). Associations among diffusion tensor image along the perivascular space (DTI-ALPS), enlarged perivascular space (ePVS), and cognitive functions in asymptomatic patients with carotid plaque. *Front. Neurol.* 12:789918. 10.3389/fneur.2021.789918 35082748 PMC8785797

[B73] Liu P. Jill B. Lu H. (2019). Cerebrovascular reactivity (CVR) MRI with CO2 challenge: A technical review. *Neuroimage* 187 104–115. 29574034 10.1016/j.neuroimage.2018.03.047 PMC6150860

[B74] Liu X. Y. Ma G. Y. Wang S. Gao Q. Guo C. Wei Q. (2022). Perivascular space is associated with brain atrophy in patients with multiple sclerosis. *Quant. Imaging Med. Surg.* 12 1004–1019. 35111601 10.21037/qims-21-705 PMC8739126

[B75] Luo X. Jiaerken Y. Yu X. Huang P. Qiu T. Jia Y. (2017). Associations between APOE genotype and cerebral small-vessel disease: A longitudinal study. *Oncotarget* 8:44477. 10.18632/oncotarget.17724 PMC5546495 28574812

[B76] Lynch M. Pham W. Sinclair B. O’Brien T. J. Law M. Vivash L. (2022). Perivascular spaces as a potential biomarker of Alzheimer’s disease. *Front. Neurosci.* 16:1021131. 10.3389/fnins.2022.1021131 36330347 PMC9623161

[B77] Manara R. Priante E. Grimaldi M. Santoro L. Astarita L. Barone R. (2011). Brain and spine MRI features of Hunter disease: Frequency, natural evolution and response to therapy. *J. Inherit. Metab. Dis.* 34 763–780. 10.1007/s10545-011-9317-5 21465231

[B78] Manara R. Rampazzo A. Cananzi M. Salviati L. Mardari R. Drigo P. (2010). Hunter syndrome in an 11-year old girl on enzyme replacement therapy with idursulfase: Brain magnetic resonance imaging features and evolution. *J. Inherit. Metab. Dis.* 33 (Suppl. 3) S67–S72. 10.1007/s10545-009-9023-8 20052546

[B79] McArdle D. J. T. Lovell T. J. H. Lekgabe E. Gaillard F. (2020). Opercular perivascular cysts: A proposed new subtype of dilated perivascular spaces. *Eur. J. Radiol.* 124:108838. 10.1016/j.ejrad.2020.108838 31972365

[B80] Mehta S. H. Nichols F. T. III Espay A. J. Duker A. P. Morgan J. C. Sethi K. D. (2013). Dilated Virchow-Robin spaces and parkinsonism. *Mov. Disord.* 28 589–590. 23575640 10.1002/mds.25474

[B81] Mestre H. Du T. Sweeney A. M. Liu G. Samson A. J. Peng W. (2020). Cerebrospinal fluid influx drives acute ischemic tissue swelling. *Science* 367:eaax7171. 10.1126/science.aax7171 32001524 PMC7375109

[B82] Mikami T. Tamada T. Suzuki H. Ukai R. Wanibuchi M. Mikuni N. (2018). Influence of hemodynamics on enlarged perivascular spaces in atherosclerotic large vessel disease. *Neurol. Res.* 40 1021–1027. 10.1080/01616412.2018.1509827 30156508

[B83] Moher D. Shamseer L. Clarke M. Ghersi D. Liberati A. Petticrew M. (2015). Preferred reporting items for systematic review and meta-analysis protocols (PRISMA-P) 2015 statement. *Syst. Rev.* 4:1. 10.1186/2046-4053-4-1 PMC4320440 25554246

[B84] Mortensen K. N. Sanggaard S. Mestre H. Lee H. Kostrikov S. Xavier A. L. (2019). Impaired glymphatic transport in spontaneously hypertensive rats. *J. Neurosci.* 39 6365–6377. 31209176 10.1523/JNEUROSCI.1974-18.2019 PMC6687896

[B85] Moses J. Sinclair B. Law M. O’Brien T. J. Vivash L. (2022). Automated methods for detecting and quantitation of enlarged perivascular spaces on MRI. *J. Magn. Reson. Imaging.* 10.1002/jmri.28369 35866259 PMC10083963

[B86] Ogawa T. Okudera T. Fukasawa H. Hashimoto M. Inugami A. Fujita H. (1995). Unusual widening of Virchow-Robin spaces: MR appearance. *Am. J. Neuroradiol.* 16 1238–1242. 7677015 PMC8337819

[B87] Ouzzani M. Hammady H. Fedorowicz Z. Elmagarmid A. (2016). Rayyan—a web and mobile app for systematic reviews. *Syst. Rev.* 5:210. 10.1186/s13643-016-0384-4 27919275 PMC5139140

[B88] Papayannis C. E. Saidon P. Rugilo C. A. Hess D. Rodriguez G. Sica R. E. (2003). Expanding Virchow Robin spaces in the midbrain causing hydrocephalus. *Am. J. Neuroradiol.* 24 1399–1403. 12917137 PMC7973669

[B89] Park S. H. Zong X. Gao Y. Lin W. Shen D. (2016). Segmentation of perivascular spaces in 7 T MR image using auto-context model with orientation-normalized features. *Neuroimage* 134 223–235. 10.1016/j.neuroimage.2016.03.076 27046107 PMC4912922

[B90] Perosa V. Oltmer J. Munting L. P. Freeze W. M. Auger C. A. Scherlek A. A. (2022). Perivascular space dilation is associated with vascular amyloid-β accumulation in the overlying cortex. *Acta Neuropathol.* 143 331–348. 10.1007/s00401-021-02393-1 34928427 PMC9047512

[B91] Piantino J. Boespflug E. L. Schwartz D. L. Luther M. Morales A. M. Lin A. (2020). Characterization of MR imaging-visible perivascular spaces in the white matter of healthy adolescents at 3T. *Am. J. Neuroradiol.* 41 2139–2145. 10.3174/ajnr.A6789 33033050 PMC7658833

[B92] Potter G. M. Doubal F. N. Jackson C. A. Chappell F. M. Sudlow C. L. Dennis M. S. (2015b). Enlarged perivascular spaces and cerebral small vessel disease. *Int. J. Stroke* 10 376–381. 23692610 10.1111/ijs.12054 PMC4463944

[B93] Potter G. M. Chappell F. M. Morris Z. Wardlaw J. M. (2015a). Cerebral perivascular spaces visible on magnetic resonance imaging: Development of a qualitative rating scale and its observer reliability. *Cerebrovasc. Dis.* 39 224–231. 10.1159/000375153 25823458 PMC4386144

[B94] Ramirez J. Berezuk C. McNeely A. A. Scott C. J. Gao F. Black S. E. (2015). Visible Virchow-Robin spaces on magnetic resonance imaging of Alzheimer’s disease patients and normal elderly from the Sunnybrook Dementia Study. *J. Alzheimers Dis.* 43 415–424. 10.3233/JAD-132528 25096616

[B95] Raposo N. Planton M. Payoux P. Péran P. Albucher J. F. Calviere L. (2019). Enlarged perivascular spaces and florbetapir uptake in patients with intracerebral hemorrhage. *Eur. J. Nucl. Med. Mol. Imaging* 46 2339–2347. 10.1007/s00259-019-04441-1 31359110

[B96] Riba-Llena I. Nafría C. Mundet X. ópez-Rueda A. L. Fernández-Cortiñas I. Jarca C. I. (2016). Assessment of enlarged perivascular spaces and their relation to target organ damage and mild cognitive impairment in patients with hypertension. *Eur. J. Neurol.* 23 1044–1050. 10.1111/ene.12979 26968973

[B97] Rivet A. Gauthier A. S. Chatain M. Billon-Grand R. Thines L. Delbosc B. (2017). A Giant tumefactive Virchow-Robin space: A rare cause of a homonymous quadrantanopia. *J. Neuroophthalmol.* 37 75–76. 10.1097/WNO.0000000000000478 28059864

[B98] Robin C. (1859). Recherches sur quelques particularites de la structure des capillaires de l’encephale. *J. Physiol. Homme. Anim.* 2 537–548.

[B99] Rocha S. Pinho J. Rito M. Machado Á (2013). Expanding Virchow-Robin spaces: Transient global amnesia and obstructive hydrocephalus. *J. Neuropsychiatry Clin. Neurosci.* 25 E49–E50. 10.1176/appi.neuropsych.12050123 23686063

[B100] Ruchoux M. M. Kalaria R. N. Román G. C. (2021). The pericyte: A critical cell in the pathogenesis of CADASIL. *Cereb. Circ. Cogn. Behav.* 2:100031. 10.1016/j.cccb.2021.100031 PMC8661128 34950895

[B101] Sahin N. Solak A. Genc B. Akpinar M. B. (2015). Dilatation of the Virchow-Robin spaces as an indicator of unilateral carotid artery stenosis: Correlation with white matter lesions. *Acta Radiol.* 56 852–859. 10.1177/0284185114544243 25140058

[B102] Salzman K. L. Osborn A. G. House P. Jinkins J. R. Ditchfield A. Cooper J. A. (2005). Giant tumefactive perivascular spaces. *Am. J. Neuroradiol.* 26 298–305. 15709127 PMC7974083

[B103] Sankararaman S. Velayuthan S. Ambekar S. Gonzalez-Toledo E. (2013). Giant tumefactive perivascular spaces: A further case. *J. Pediatr. Neurosci.* 8 108–110. 24082925 10.4103/1817-1745.117837 PMC3783714

[B104] Sati P. Oh J. Constable R. T. Evangelou N. Guttmann C. R. Henry R. G. (2016). The central vein sign and its clinical evaluation for the diagnosis of multiple sclerosis: A consensus statement from the North American Imaging in Multiple Sclerosis Cooperative. *Nat. Rev. Neurol.* 12 714–722. 10.1038/nrneurol.2016.166 27834394

[B105] Sawada M. Nishi S. Hashimoto N. (1999). Unilateral appearance of markedly dilated Virchow-Robin spaces. *Clin. Radiol.* 54 334–336. 10.1016/s0009-9260(99)90566-4 10362243

[B106] Sepehrband F. Barisano G. Sheikh-Bahaei N. Cabeen R. P. Choupan J. Law M. (2019). Image processing approaches to enhance perivascular space visibility and quantification using MRI. *Sci. Rep.* 9 1–12. 10.1038/s41598-019-48910-x 31451792 PMC6710285

[B107] Sepehrband F. Barisano G. Sheikh-Bahaei N. Choupan J. Cabeen R. P. Lynch K. M. (2021). Volumetric distribution of perivascular space in relation to mild cognitive impairment. *Neurobiol. Aging* 99 28–43. 10.1016/j.neurobiolaging.2020.12.010 33422892 PMC7902350

[B108] Shams S. Martola J. Charidimou A. Cavallin L. Granberg T. Shams M. (2016). Cortical superficial siderosis: Prevalence and biomarker profile in a memory clinic population. *Neurology* 87 1110–1117. 10.1212/WNL.0000000000003088 27534713 PMC5027806

[B109] Sim J. E. Park M. S. Shin H. Y. Jang H. S. Won H. H. Seo S. W. (2020). Correlation between hippocampal enlarged perivascular spaces and cognition in non-dementic elderly population. *Front. Neurol.* 11:542511. 10.3389/fneur.2020.542511 33133000 PMC7550712

[B110] Song Q. Cheng Y. Wang Y. Liu J. Wei C. Liu M. (2021). Enlarged perivascular spaces and hemorrhagic transformation after acute ischemic stroke. *Ann. Transl. Med.* 9:1126. 10.21037/atm-21-1276 PMC8350705 34430567

[B111] Spangler K. M. Challa V. R. Moody D. M. Bell M. A. (1994). Arteriolar tortuosity of the white matter in aging and hypertension. A microradiographic study. *J. Neuropathol. Exp. Neurol.* 53 22–26. 10.1097/00005072-199401000-00003 8301316

[B112] Spijkerman J. Zwanenburg J. Bouvy W. Geerlings M. Biessels G. Hendrikse J. (2022). Automatic quantification of perivascular spaces in T2-weighted images at 7 T MRI. *Cereb. Circ. Cogn. Behav.* 3:100142. 10.1016/j.cccb.2022.100142 36324395 PMC9616283

[B113] Taniguchi D. Shimura H. Watanabe M. Hattori N. Urabe T. (2017). Widespread enlarged perivascular spaces associated with dementia and focal brain dysfunction: Case report. *BMC Neurol.* 17:210. 10.1186/s12883-017-0997-9 29212461 PMC5719666

[B114] Taoka T. Masutani Y. Kawai H. Nakane T. Matsuoka K. Yasuno F. (2017). Evaluation of glymphatic system activity with the diffusion MR technique: Diffusion tensor image analysis along the perivascular space (DTI-ALPS) in Alzheimer’s disease cases. *Jpn. J. Radiol.* 35 172–178. 10.1007/s11604-017-0617-z 28197821

[B115] Thore C. R. Anstrom J. A. Moody D. M. Challa V. R. Marion M. C. Brown W. R. (2007). Morphometric analysis of arteriolar tortuosity in human cerebral white matter of preterm, young, and aged subjects. *J. Neuropathol. Exp. Neurol.* 66 337–345. 10.1097/nen.0b013e3180537147 17483690

[B116] Troili F. Cipollini V. Moci M. Morena E. Palotai M. Rinaldi V. (2020). Perivascular unit: This must be the place. the anatomical crossroad between the immune, vascular and nervous system. *Front. Neuroanat.* 14:17. 10.3389/fnana.2020.00017 32372921 PMC7177187

[B117] Tsai H. H. Lee B. C. Chen Y. F. Jeng J. S. Tsai L. K. (2022). Cerebral venous reflux and dilated basal ganglia perivascular space in hypertensive intracerebral hemorrhage. *J. Stroke* 24 363–371. 10.5853/jos.2022.01004 36221939 PMC9561214

[B118] Tsai H. H. Pasi M. Tsai L. K. Huang C. C. Chen Y. F. Lee B. C. (2021). Centrum semiovale perivascular space and amyloid deposition in spontaneous intracerebral hemorrhage. *Stroke* 52 2356–2362. 10.1161/STROKEAHA.120.032139 33874751 PMC8989045

[B119] Tseng H. S. Ho C. S. Chiu N. C. (2013). Multiple giant Virchow-Robin spaces. *Pediatr. Neurol.* 49:143. 10.1016/j.pediatrneurol.2013.01.006 23683658

[B120] Tsutsumi S. Ito M. Yasumoto Y. Tabuchi T. Ogino I. (2011). The Virchow-Robin spaces: Delineation by magnetic resonance imaging with considerations on anatomofunctional implications. *Childs Nerv. Syst.* 27 2057–2066. 10.1007/s00381-011-1574-y 21909964

[B121] Ungvari Z. Toth P. Tarantini S. Prodan C. I. Sorond F. Merkely B. (2021). Hypertension-induced cognitive impairment: From pathophysiology to public health. *Nat. Rev. Nephrol.* 17 639–654. 34127835 10.1038/s41581-021-00430-6 PMC8202227

[B122] Van Veluw S. J. Biessels G. J. Bouvy W. H. Spliet W. G. Zwanenburg J. J. Luijten P. R. (2016). Cerebral amyloid angiopathy severity is linked to dilation of juxtacortical perivascular spaces. *J. Cereb. Blood Flow Metab.* 36 576–580. 10.1177/0271678X15620434 26661250 PMC4794108

[B123] Vikner T. Karalija N. Eklund A. Malm J. Lundquist A. Gallewicz N. (2022). 5-year associations among cerebral arterial pulsatility, perivascular space dilation, and white matter lesions. *Ann. Neurol.* 92 871–881. 10.1002/ana.26475 36054261 PMC9804392

[B124] Virchow R. (1851). Ueber die erweiterung kleinerer gefässe. *Virchows Archiv.* 3 427–462.

[B125] Wang M.-L. Yu M.-M. Wei X.-E. Li W.-B. Li Y.-H. Alzheimer’s Disease Neuroimaging Initiative (2021). Association of enlarged perivascular spaces with Aβ and tau deposition in cognitively normal older population. *Neurobiol. Aging* 100 32–38. 10.1016/j.neurobiolaging.2020.12.014 33477009

[B126] Wang X. X. Cao Q. C. Teng J. F. Wang R. F. Yang Z. T. Wang M. G. (2022). MRI-visible enlarged perivascular spaces: Imaging marker to predict cognitive impairment in older chronic insomnia patients. *Eur. Radiol.* 32 5446–5457. 35286409 10.1007/s00330-022-08649-y

[B127] Wang X. Feng H. Wang Y. Zhou J. Zhao X. (2019). Enlarged perivascular spaces and cerebral small vessel disease in spontaneous intracerebral hemorrhage patients. *Front. Neurol.* 10:881. 10.3389/fneur.2019.00881 31474932 PMC6702269

[B128] Wang X. Valdes Hernandez Mdel C. Doubal F. Chappell F. M. Piper R. J. Deary I. J. (2016). Development and initial evaluation of a semi-automatic approach to assess perivascular spaces on conventional magnetic resonance images. *J. Neurosci. Methods* 257 34–44. 10.1016/j.jneumeth.2015.09.010 26416614 PMC4666413

[B129] Wardlaw J. M. Benveniste H. Nedergaard M. Zlokovic B. V. Mestre H. Lee H. (2020). Perivascular spaces in the brain: Anatomy, physiology and pathology. *Nat. Rev. Neurol.* 16 137–153. 10.1038/s41582-020-0312-z 32094487

[B130] Wardlaw J. M. Doubal F. Armitage P. Chappell F. Carpenter T. Muñoz Maniega S. (2009). Lacunar stroke is associated with diffuse blood–brain barrier dysfunction. *Ann. Neurol.* 65 194–202. 10.1002/ana.21549 19260033

[B131] Wardlaw J. M. Smith E. E. Biessels G. J. Cordonnier C. Fazekas F. Frayne R. (2013a). Neuroimaging standards for research into small vessel disease and its contribution to ageing and neurodegeneration. *Lancet Neurol.* 12 822–838. 10.1016/S1474-4422(13)70124-8 23867200 PMC3714437

[B132] Wardlaw J. M. Smith C. Dichgans M. (2013b). Mechanisms of sporadic cerebral small vessel disease: Insights from neuroimaging. *Lancet Neurol.* 12 483–497. 10.1016/S1474-4422(13)70060-7 23602162 PMC3836247

[B133] Williamson B. J. Khandwala V. Wang D. Maloney T. Sucharew H. Horn P. (2022). Automated grading of enlarged perivascular spaces in clinical imaging data of an acute stroke cohort using an interpretable, 3D deep learning framework. *Sci. Rep.* 12 1–7. 10.1038/s41598-021-04287-4 35039524 PMC8764081

[B134] Wiseman R. Saxby B. Burton E. Barber R. Ford G. O’brien J. (2004). Hippocampal atrophy, whole brain volume, and white matter lesions in older hypertensive subjects. *Neurology* 63 1892–1897. 10.1212/01.wnl.0000144280.59178.78 15557507

[B135] Wooliscroft L. Boespflug E. Hildebrand A. Shangraw K. Silbermann E. Bourdette D. (2020). Enlarged perivascular spaces are not associated with vascular co-morbidities, clinical outcomes, and brain volumes in people with secondary progressive multiple sclerosis. *Mult. Scler. J. Exp. Transl. Clin.* 6:2055217320964502. 10.1177/2055217320964502 33110618 PMC7557790

[B136] Wostyn P. Mader T. H. Gibson C. R. Nedergaard M. (2022a). Does long-duration exposure to microgravity lead to dysregulation of the brain and ocular glymphatic systems? *Eye Brain* 14:49. 10.2147/EB.S354710 35546965 PMC9081191

[B137] Wostyn P. Mader T. H. Gibson C. R. Nedergaard M. (2022b). The effect of long-duration spaceflight on perivascular spaces within the brain. *Proc. Natl. Acad. Sci. U.S.A.* 119 e2207724119. 10.1073/pnas.2207724119 PMC9371637 35858378

[B138] Wuerfel J. Haertle M. Waiczies H. Tysiak E. Bechmann I. Wernecke K. D. (2008). Perivascular spaces–MRI marker of inflammatory activity in the brain? *Brain* 131 2332–2340. 10.1093/brain/awn171 18676439

[B139] Xia Y. Shen Y. Wang Y. Yang L. Wang Y. Li Y. (2020). White matter hyperintensities associated with progression of cerebral small vessel disease: A 7-year Chinese urban community study. *Aging* 12 8506–8522. 10.18632/aging.103154 32388497 PMC7244059

[B140] Xu J. Su Y. Fu J. Wang X. Nguchu B. A. Qiu B. (2022). Glymphatic dysfunction correlates with severity of small vessel disease and cognitive impairment in cerebral amyloid angiopathy. *Eur. J. Neurol.* 29 2895–2904. 10.1111/ene.15450 35712978

[B141] Yamaguchi Y. Wada M. Kimihira L. Nagasawa H. (2021). Cognitive impairment due to widespread enlarged perivascular spaces. *Radiol. Case Rep.* 16 2640–2645. 10.1016/j.radcr.2021.06.043 34345324 PMC8319478

[B142] Yu N. Sinclair B. Posada L. M. G. Chen Z. Di Q. Lin X. (2022). Asymmetric distribution of enlarged perivascular spaces in centrum semiovale may be associated with epilepsy after acute ischemic stroke. *CNS Neurosci. Ther.* 28 343–353. 10.1111/cns.13786 34981639 PMC8841310

[B143] Zhang K. Zhou Y. Zhang W. Li Q. Sun J. Lou M. (2022). MRI-visible perivascular spaces in basal ganglia but not centrum semiovale or hippocampus were related to deep medullary veins changes. *J. Cereb. Blood Flow Metab.* 42 136–144. 10.1177/0271678X211038138 34431378 PMC8721776

[B144] Zhang X. Ding L. Yang L. Qin W. Yuan J. Li S. (2016). Brain atrophy correlates with severe enlarged perivascular spaces in basal ganglia among lacunar stroke patients. *PLoS One* 11:e0149593. 10.1371/journal.pone.0149593 26900696 PMC4764761

[B145] Zhu Y.-C. Dufouil C. Mazoyer B. Soumaré A. Ricolfi F. Tzourio C. (2011). Frequency and location of dilated Virchow-Robin spaces in elderly people: A population-based 3D MR imaging study. *Am. J. Neuroradiol.* 32 709–713. 10.3174/ajnr.A2366 21349956 PMC7965873

[B146] Zong X. Lian C. Jimenez J. Yamashita K. Shen D. Lin W. (2020). Morphology of perivascular spaces and enclosed blood vessels in young to middle-aged healthy adults at 7T:Dependences on age, brain region, and breathing gas. *Neuroimage* 218:116978. 10.1016/j.neuroimage.2020.116978 32447015 PMC7485170

[B147] Zou Q. Wang M. Wei X. Li W. (2022). Prevalence and risk factors for enlarged perivascular spaces in young adults from a neurology clinic-based cohort. *Brain Sci.* 12:1164. 10.3390/brainsci12091164 36138900 PMC9497082

